# Cyclodextran prevents *Porphyromonas gulae* and *Porphyromonas gingivalis* induced halitosis and cytokine secretion via direct inhibition of biofilm formation

**DOI:** 10.3389/froh.2025.1713668

**Published:** 2025-12-18

**Authors:** Megu Toyooka, Mao Kaneki, Chiharu Ohira, Yasuyuki Nakamura, Mio Yamamoto, Tomoki Fukuyama

**Affiliations:** 1 Laboratory of Veterinary Pharmacology, School of Veterinary Medicine, Azabu University, Sagamihara-shi, Japan; 2 WELLNEO SUGAR Co., Ltd., Chuo-ku, Japan; 3 Center for Human and Animal Symbiosis Science, Azabu University, Sagamihara-shi, Japan

**Keywords:** cyclodextran, periodontal disease, porphyromonas gulae, porphyromonas gingivalis, biofilm

## Abstract

Periodontal disease (PD) is an inflammatory condition affecting the supporting structures of teeth, initiated by bacterial biofilm formation. *Porphyromonas gulae* (*P. gulae*) and *P. gingivalis* are key pathogens in canine and human PD, respectively, producing biofilms, volatile sulfur compounds, and proinflammatory cytokines that contribute to halitosis and tissue destruction. Cyclodextran (CI), a cyclic oligosaccharide, has previously been shown to inhibit glucan synthesis in Streptococcus mutans, but its effects on periodontal bacteria remain unexplored. This study investigated the influence of CI on *P. gulae* and *P. gingivalis in vitro*. Bacterial cultures were co-incubated with varying concentrations of a CI-dextran mix (0.313%–5%) for up to 24 h. Biofilm formation and insoluble glucan production were assessed via fluorescence microscopy and biochemical assays. Hydrogen sulfide and methyl mercaptan levels were measured by gas chromatography, and cytokine production (IL-1β, IL-6) was quantified in murine and canine macrophage cell lines using ELISA. CI had limited bactericidal activity but significantly inhibited biofilm formation and glucan production in both bacterial species. Consequently, hydrogen sulfide and methyl mercaptan generation were markedly reduced, although CI did not directly neutralize these compounds. Furthermore, CI treatment significantly suppressed *P. gulae* and *P. gingivalis*-induced IL-1β and IL-6 secretion in macrophages in a dose-dependent manner without cytotoxicity. These findings demonstrate that cyclodextran prevents PD-related halitosis and inflammation primarily by inhibiting biofilm formation rather than bacterial killing or direct deodorization. CI represents a promising candidate for preventive oral care in humans and companion animals, with potential to reduce the onset and progression of PD.

## 1 Introduction

Periodontal disease (PD) is a common inflammatory disorder affecting the supporting structures of the teeth, including the gingiva, periodontal ligament, and alveolar bone. It is initiated by bacterial colonization and biofilm formation on the tooth surface, which triggers local inflammation and progressive tissue destruction. If left untreated, PD can lead to irreversible gingival recession, alveolar bone loss, tooth mobility, and eventual tooth loss. PD is prevalent in both humans and companion animals, particularly dogs, making it a significant concern in veterinary and human dental medicine (1, 2).

The initial stage of PD involves the formation of a biofilm, commonly referred to as dental plaque, on tooth surfaces. This biofilm is composed of extracellular polysaccharides, primarily glucans, produced by periodontal bacteria. Biofilm formation not only promotes bacterial adherence and colonization but also shields bacteria from host immune responses and reduces the efficacy of antibiotics (3). Among the key pathogens implicated in PD are *Porphyromonas gingivalis* (*P. gingivalis*) in humans and *Porphyromonas gulae* (*P. gulae*) in dogs. Both species belong to the “red complex” bacteria, which are strongly associated with the progression and severity of PD (4). These bacteria produce virulence factors, including hydrogen sulfide and methyl mercaptan, which contribute to halitosis, tissue inflammation, and periodontal tissue degradation. Additionally, these bacteria stimulate the secretion of proinflammatory cytokines such as interleukin (IL)-1β and IL-6 from host immune cells, further exacerbating periodontal inflammation (5, 6).

Cyclodextran (CI) is a cyclic oligosaccharide composed of 7–12 glucose units, typically produced by *Bacillus* species (7). Although its name resembles that of cyclodextrins, the two compounds differ markedly in both structure and biochemical properties. Cyclodextrins are cyclic α-1,4-linked glucose oligomers known for their hydrophobic cavities, which enable them to encapsulate small molecules and serve primarily as inclusion complexes. In contrast, cyclodextrans are cyclic α-1,6-linked isomaltooligosaccharides. This structural difference imparts unique biochemical properties, including the ability to interact with glucosyltransferases (Gtf) and inhibit α-1,6-glucan synthesis. Previous studies indicate that CI suppresses Gtf activity primarily through direct interaction with the enzyme's acceptor site, competitively blocking α-1,6-glucan chain elongation rather than sequestering substrates, thereby inhibiting glucan-dependent biofilm formation in *Streptococcus mutans* (8). Because *P. gulae* and *P. gingivalis* also rely on extracellular polysaccharide synthesis for biofilm stability, we hypothesized that CI could similarly disrupt glucan-mediated biofilm formation in these periodontal pathogens. This represents a novel mechanistic extension of CI's activity beyond cariogenic streptococci to *Porphyromonas* species implicated in PD.

Given the critical role of biofilm formation in PD pathogenesis, targeting biofilm development represents a promising strategy for disease prevention. This study aimed to evaluate the direct effects of CI on biofilm formation, halitosis-related compound production, and proinflammatory cytokine induction by *P. gulae* and *P. gingivalis in vitro*. By elucidating these effects, we sought to explore the potential of CI as a preventive agent for PD in both humans and companion animals.

## 2 Methods

### 2.1 Bacterial strains and culture conditions

*P. gulae* ATCC 51700 (fimA-type) and *P. gingivalis* ATCC 2561 were obtained from the Japan Collection of Microorganisms (RIKEN BioResource Research Center, Tokyo, Japan). Both strains were grown anaerobically at 37°C for 72 h on BD BBL™ CDC Anaerobe 5% Sheep Blood Agar (Becton, Dickinson and Company, Franklin Lakes, NJ, USA) and horse red cell contained Brucella broth (KYOKUTO PHARMACEUTICAL INDUSTRIAL CO., LTD., Tokyo, Japan). Bacterial inocula were adjusted to an optical density at 600 nm (OD₆₀₀) corresponding to approximately 4 × 10⁸ CFU/mL, which reflects clinically relevant bacterial loads reported in subgingival plaque of dogs with moderate-to-severe PD (9, 10). Inocula were prepared in the same manner for all experiments, and equivalent OD₆₀₀ values were used to ensure comparability across replicates. This concentration was selected to reflect clinically relevant bacterial loads reported in subgingival plaque.

### 2.2 Cyclodextran preparation

Cyclodextran (CI)–dextran mix was kindly provided by WELLNEO SUGAR Co., Ltd. (Tokyo, Japan). The formulation contained >13% cyclic isomaltooligosaccharides (cyclodextran) and linear dextran as carrier polysaccharides. The CI–dextran mix was stored in a desiccated state at room temperature until use. The CI–dextran mix provided adequate solubility and stability while minimizing changes in viscosity or surface properties that could otherwise influence bacterial adhesion. This composition was selected to ensure that the observed inhibitory effects primarily reflect the functional properties of CI rather than nonspecific contributions from dextran. For experimental assays, the CI–dextran mix was freshly dissolved in the appropriate culture medium to achieve final concentrations of 0.313%, 0.625%, 1.25%, 2.5%, and 5% (w/v). The concentration range of the CI–dextran mix was determined based on preliminary dose-ranging experiments conducted to evaluate biofilm inhibition and cytotoxicity. In these pilot assays, CI began to inhibit biofilm formation at approximately 0.3%, with increasing inhibition observed up to 5%, beyond which no further enhancement was detected and solubility decreased. Cytotoxicity testing using mammalian cell lines (RAW264.7 and DH82) showed no significant reduction in cell viability within this range (data not shown). The selected concentrations were therefore used to represent sub-effective, intermediate, and maximal non-cytotoxic doses. In addition, the study by Kobayashi, Funane (8) was referenced, as it reported comparable effective concentrations of cyclodextran for inhibiting *Streptococcus mutans* Gtf activity, further supporting the chosen range.

### 2.3 Bacterial viability assay

The bactericidal effect of CI on *P. gulae* and *P. gingivalis* was evaluated using the BacTiter-Glo™ Microbial Cell Viability Assay (Promega KK, Tokyo, Japan), which quantifies metabolically active cells through ATP-dependent luminescence. Bacterial suspensions (1 × 10⁷ CFU/mL) were incubated with CI at final concentrations of 0.313%, 0.625%, 1.25%, 2.5%, and 5% (w/v) for 10 min or 1 h under anaerobic conditions at 37 °C. After incubation, 100 µL of each bacterial suspension was mixed with an equal volume of BacTiter-Glo reagent in a 96-well white plate and gently agitated for 1 min at room temperature. Luminescence was measured using a GloMax® Multi Detection System (Promega KK). A decrease in luminescence indicated loss of metabolic activity consistent with a bactericidal effect. Negative controls consisted of untreated bacteria incubated under identical conditions. Each concentration was tested in eight technical replicates, and all experiments were independently repeated three times.

### 2.4 Biofilm formation assay

Biofilm formation was evaluated using a Biofilm Formation Assay Kit (Dojindo Laboratories, Kumamoto, Japan) according to the manufacturer's instructions. Briefly, bacterial suspensions of *P. gulae* or *P. gingivalis* were incubated with various concentrations (0.313%, 0.625%, 1.25%, 2.5%, 5%) of CI–dextran mix in 96-well plates for 24 h under anaerobic conditions at 37 °C. After incubation, non-adherent cells were gently removed by washing twice with phosphate-buffered saline. Biofilms were stained with crystal violet, washed, and destained, and absorbance was measured at 595 nm using a microplate reader. The degree of biofilm inhibition was calculated relative to untreated controls. To visualize biofilm structure and cell viability, biofilms formed on glass coverslips were examined by scanning electron microscopy (SEM) using a Miniscope® TM4000Plus III (Hitachi High-Tech Corporation, Tokyo, Japan) and by fluorescence microscopy (BZ-X800, Keyence Corporation, Tokyo, Japan) following staining with the FilmTracer™ LIVE/DEAD® Biofilm Viability Kit (Thermo Fisher Scientific, Waltham, MA, USA). SEM images were used to assess surface morphology, whereas fluorescence microscopy distinguished viable (green) and non-viable (red) cells within the biofilm matrix. Each experimental condition was tested in eight technical replicates and repeated independently three times.

### 2.5 Insoluble glucan quantification

To evaluate the effect of CI on extracellular biofilm matrix components, insoluble glucan (mutan) production was quantified. Overnight cultures of *P. gulae and P. gingivalis* were co-incubated with CI–dextran mix at final concentrations of 0.625%, 1.25%, 2.5%, and 5% (w/v) in brain heart infusion broth supplemented with 1% glucose for 24 h under anaerobic conditions at 37°C. After incubation, cultures were centrifuged at 10,000 × g for 10 min, and the supernatants were discarded. The remaining pellets containing insoluble glucans were washed twice with PBS and hydrolyzed with 0.1 M NaOH for 1 h at room temperature. The amount of released carbohydrate was determined using the phenol–sulfuric acid method (11), with D-glucose as a standard. Absorbance was measured at 490 nm using a microplate reader. All assays were performed in triplicate and independently repeated three times.

### 2.6 Volatile sulfur compound (VSC) analysis

Hydrogen sulfide (H₂S) and methyl mercaptan (CH₃SH) production were quantified as biochemical markers of halitosis. Bacterial cultures of *P. gulae* and *P. gingivalis* were co-incubated with CI–dextran mix at final concentrations of 0.625%, 1.25%, 2.5%, and 5% (w/v) for 24 h under anaerobic conditions at 37 °C. After incubation, 1 mL of headspace gas from each culture tube was collected using a gas-tight syringe. Concentrations of H₂S and CH₃SH were determined by gas chromatography (OralChroma™, Nissha FIS Inc., Tokyo, Japan), a compact GC system optimized for oral malodor analysis. This instrument contains a built-in column and utilizes hydrogen as the carrier gas under fixed flow and temperature conditions specified by the manufacturer. Results were expressed as parts per billion (ppb). To examine whether CI directly neutralizes sulfur compounds, a chemical deodorization assay was performed using a methyl mercaptan standard solution (FUJIFILM Wako Pure Chemical Corporation, Osaka, Japan). CI–dextran mix (0.625%–5%) was incubated with the standard for 10 min at room temperature, and residual CH₃SH concentrations were quantified using the same gas chromatography conditions. Each condition was analyzed in triplicate, and all experiments were repeated independently three times.

### 2.7 Cytokine production assay

The effects of CI on *P. gulae* and *P. gingivalis*-induced inflammatory responses were evaluated using murine macrophage RAW264.7 (Japanese Collection of Research Bioresources, Tokyo, Japan) and canine macrophage DH82 cell lines (American Type Culture Collection, Manassas, VA, USA). Cells were cultured in Roswell Park Memorial Institute (RPMI) 1,640 medium or Eagle's minimum essential medium (EMEM; FUJIFILM Wako Pure Chemical Corporation, Osaka, Japan) supplemented with 10% fetal bovine serum (FBS) and 1% penicillin–streptomycin at 37 °C in a humidified atmosphere containing 5% CO₂. For stimulation assays, macrophages were seeded in 96-well plates at a density of 1 × 10^4^ cells/well and allowed to adhere overnight. *P. gulae* or *P. gingivalis* were added to the macrophage cultures in the presence or absence of CI–dextran mix at final concentrations of 0.625%, 1.25%, 2.5%, and 5% (w/v). After 24 h of incubation, culture supernatants were collected. IL-1β and IL-6 concentrations were quantified using DuoSet® ELISA kits (R&D Systems, Minneapolis, MN, USA) according to the manufacturer's instructions. Absorbance was measured at 450 nm using a microplate reader, and cytokine concentrations were calculated based on standard curves. Cytotoxicity was evaluated by monitoring cell viability under the same treatment conditions. Each experimental condition was tested in eight technical replicates and repeated independently three times.

### 2.8 Statistical analysis

All quantitative data are expressed as the mean ± standard error of the mean (SEM). Prior to applying parametric tests, data were evaluated for normality and equal variances. Equality of variances was assessed using the Brown–Forsythe test, and homogeneity of variances was verified with Bartlett's test. Only datasets satisfying these assumptions were analyzed using one-way ANOVA followed by Dunnett's multiple-comparison test to compare each treatment group with the control. Statistical analyses were performed using GraphPad Prism version 10.0 (GraphPad Software, San Diego, CA, USA), and a *p*-value of <0.05 was considered statistically significant.

## 3 Results

### 3.1 CI exhibits anti-biofilm but not antimicrobial activity

**Figure 1:**
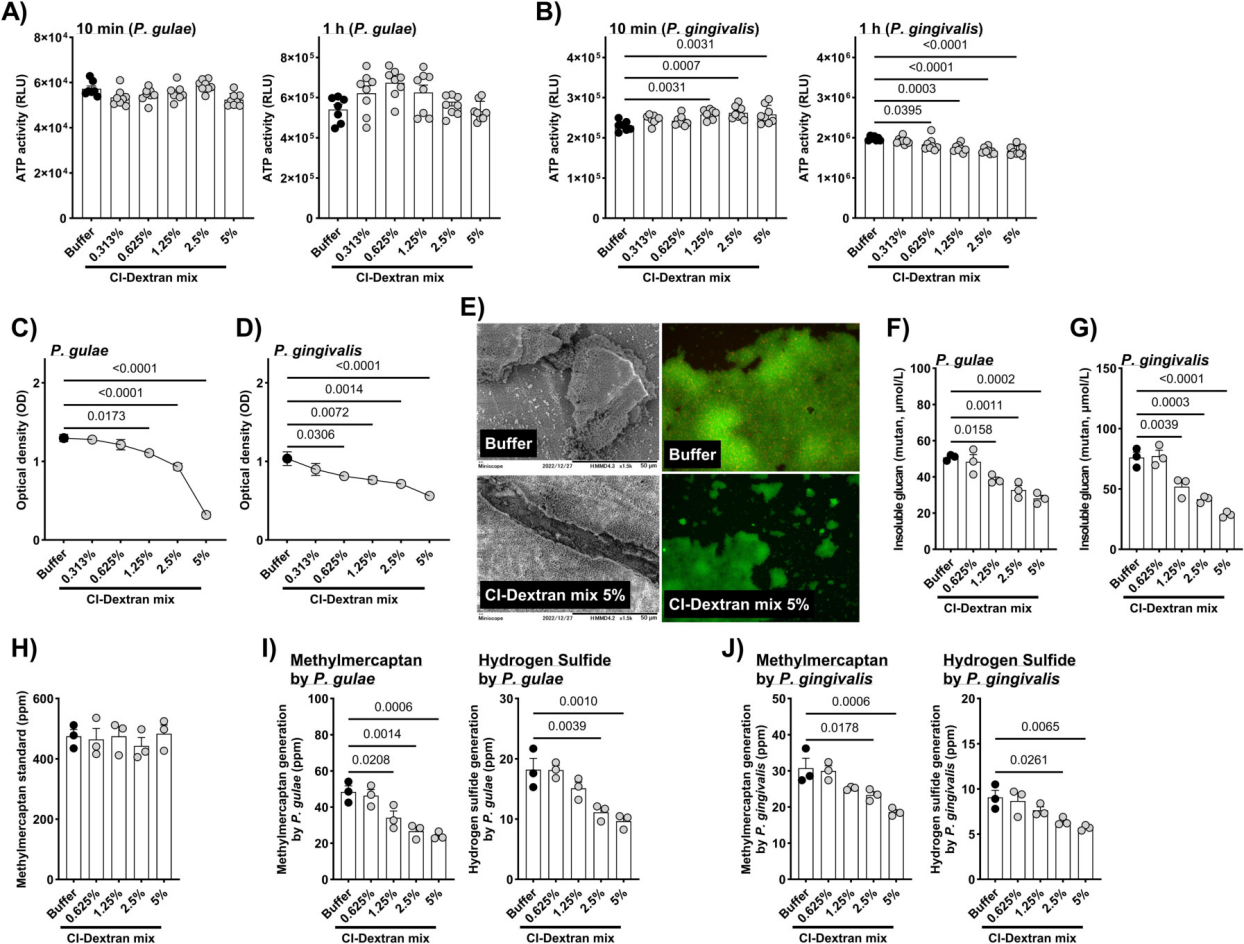
Influence of CI-dextran mix on bacterial growth, biofilm formation, insoluble glucan production, and volatile sulfur compound generation in *P. gulae* and *P. gingivalis*. **(A,B)** Growth of *P. gulae* and *P. gingivalis* (ATP activity, RLU) was not significantly affected by CI-dextran mix treatment for 10 min to 1 h. **(C,D)** Biofilm formation was significantly inhibited after 24 h of CI-dextran mix treatment. **(E)** Representative electron microscopy and fluorescence microscopy images of *P. gulae* biofilms showing reduced density and structural disruption following CI treatment. Insoluble glucan (mutan, μmol/L) production by **(F)** *P. gulae* and **(G)** *P. gingivalis* was significantly reduced in a dose-dependent manner. **(H)** The CI-dextran mix showed no direct deodorant effect on a methyl mercaptan standard (ppm), whereas methyl mercaptan and hydrogen sulfide (ppm) produced by **(I)** *P. gulae* and **(J)** *P. gingivalis* were significantly suppressed after 24 h of treatment. Data are presented as mean ± SEM; n = 3–8 per group. *p* < 0.05 (Dunnett's multiple comparison test) vs. buffer control.

CI treatment did not significantly reduce the viability of *P. gulae* at concentrations up to 5% (w/v), as determined by the BacTiter-Glo™ assay (Figure 1A). Luminescence intensity, reflecting intracellular ATP levels, remained comparable to untreated controls after both 10 min and 1 h of exposure, indicating that CI exerts no direct bactericidal effect on *P. gulae*. In contrast, *P. gingivalis* exhibited a concentration-dependent decrease in ATP activity following 1 h of CI–dextran treatment (Figure 1B), suggesting mild metabolic inhibition at higher concentrations. However, this reduction was substantially lower than that typically observed with bactericidal agents (control: 1,980,100 ± 19,252 RLU; CI 5%: 1,674,324 ± 42,002 RLU; 15% reduction, *p* < 0.0001), implying that CI primarily suppresses bacterial metabolism rather than inducing cell death.

Despite the limited antimicrobial activity, CI markedly inhibited biofilm formation by both *P. gulae* and *P. gingivalis* in a concentration-dependent manner (Figures 1C,D). Crystal violet quantification showed significant reductions in biofilm biomass at ≥1.25% CI (*p* < 0.01), despite unchanged bacterial viability. Biofilm biomass decreased by approximately 80% at 5% CI in *P. gulae* biofilms (control = 1.30 ± 0.045; CI 5% = 0.32 ± 0.35; *p* < 0.0001). SEM analysis revealed disrupted biofilm architecture characterized by sparse bacterial clusters and diminished extracellular matrix compared with the dense, multilayered structures observed in untreated controls (Figure 1E). Live/Dead fluorescence imaging confirmed that most cells within CI-treated biofilms remained viable, further supporting that CI impairs biofilm formation and structural integrity without exhibiting bactericidal effects.

### 3.2 Reduction of insoluble glucan production

The production of insoluble glucan, a key extracellular component of the biofilm matrix, was significantly reduced in both *P. gulae* and *P. gingivalis* following CI treatment (0.625%–5% w/v) (Figures 1F,G). In *P. gulae*, insoluble glucan levels decreased from 50.7 ± 0.95 μmol/L in the control to 38.6 ± 1.37 μmol/L at 1.25% CI (*p* = 0.0158) and 28.1 ± 1.76 μmol/L at 5% CI (*p* = 0.0002), corresponding to approximately 45% reduction at the highest concentration. Similarly, in *P. gingivalis*, glucan production was reduced from 76.0 ± 4.52 μmol/L in the control to 51.9 ± 4.82 μmol/L at 1.25% CI (*p* = 0.0039) and 28.7 ± 1.39 μmol/L at 5% CI (*p* < 0.0001). The dose-dependent decline in glucan content closely paralleled the decrease in total biofilm biomass, supporting the conclusion that CI directly inhibits glucan-mediated biofilm development rather than exerting a bactericidal effect.

### 3.3 Suppression of halitosis-related compounds

Hydrogen sulfide (H₂S) and methyl mercaptan (CH₃SH), the major VSCs associated with PD–related halitosis, were markedly reduced by CI treatment in both *P. gulae* and *P. gingivalis* cultures (Figures 1H–J). After 24 h co-incubation with CI-dextran, H₂S levels in *P. gulae* decreased from 18.2 ± 1.87 ppb in controls to 9.67 ± 0.71 ppb at 5% CI (*p* = 0.001), representing an approximately 47% reduction at the highest concentration. Similarly, CH₃SH levels declined from 48.4 ± 3.35 ppb (control) to 24.2 ± 1.20 ppb at 5% CI (*p* = 0.0006). Comparable dose-dependent decreases were observed in *P. gingivalis* cultures. In contrast, CI did not directly neutralize CH₃SH when incubated with a methyl mercaptan standard for 10 min, indicating that the reduction in VSCs results from inhibition of bacterial metabolic activity rather than direct chemical deodorization.

### 3.4 Inhibition of proinflammatory cytokine production

**Figure 2:**
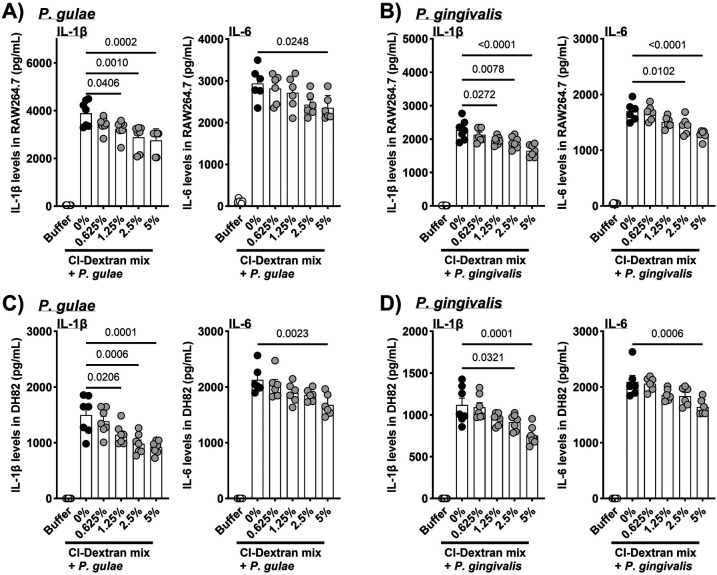
Inhibitory effects of CI-dextran mix on *P. gulae*- and *P. gingivalis*-induced proinflammatory cytokine secretion in macrophages. CI-dextran mix treatment significantly reduced the production of IL-1β (pg/mL) and IL-6 (pg/mL) in **(A,B)** murine RAW264.7 cells and **(C,D)** canine DH82 cells following bacterial stimulation. Data are presented as mean ± SEM; *n* = 7 per group. *p* < 0.05 (Dunnett's multiple comparison test) vs. untreated control. IL, interleukin.

To evaluate the anti-inflammatory potential of CI, IL-1β and IL-6 secretion was measured in murine (RAW264.7) and canine (DH82) macrophages stimulated with *P. gulae* or *P. gingivalis*. Co-culture with bacteria markedly increased cytokine production in untreated controls (*P. gingivalis*–stimulated RAW264.7: IL-1β = 2,272 ± 114 pg/mL, IL-6 = 1,698 ± 69 pg/mL). CI-dextran mix treatment significantly and dose-dependently suppressed cytokine secretion without affecting cell viability (Figures 2A–D). In RAW264.7 cells, IL-1β levels decreased to 1,657 ± 67 pg/mL at 5% CI (*p* < 0.0001), representing a 27% reduction compared with untreated controls. Similarly, IL-6 levels declined to 1,278 ± 36 pg/mL at 5% CI (*p* < 0.0001). Comparable inhibitory effects were observed in DH82 macrophages, where IL-1β and IL-6 secretion decreased by approximately 20%–30% at the highest CI concentration.

## 4 Discussion

PD is a prevalent inflammatory condition that affects the supporting structures of teeth and is characterized by bacterial biofilm formation, halitosis, and chronic inflammation (12). The pathogenesis of PD is closely associated with the “red complex” bacteria, including *P. gingivalis* in humans and *P. gulae* in companion animals (13–15). These pathogens produce extracellular polysaccharides, particularly glucans, which facilitate biofilm formation and bacterial colonization on tooth surfaces. Biofilms not only enhance bacterial persistence by providing protection from host immune responses but also reduce the efficacy of conventional antimicrobial therapies, such as tetracyclines and clindamycin (16). Furthermore, the metabolic activity of these bacteria generates volatile sulfur compounds (VSCs) like hydrogen sulfide (H₂S) and methyl mercaptan (CH₃SH), which contribute to halitosis, tissue destruction, and further inflammatory responses (17). Therefore, strategies that can inhibit biofilm formation without relying solely on bactericidal activity are highly desirable for preventing and managing PD in both humans and companion animals.

In this study, we evaluated the effects of CI, a cyclic oligosaccharide composed of 7–12 glucose units, on the biofilm formation, virulence factor production, and inflammatory responses induced by *P. gulae* and *P. gingivalis*. CI has previously been reported to inhibit glucan synthesis in *Streptococcus mutans*, reducing dental plaque formation (8). Unlike conventional cyclodextrin, which lacks significant anti-biofilm activity, CI exerts strong inhibitory effects on bacterial glucosyltransferase activity. Our results extend these observations to periodontal pathogens and demonstrate, for the first time, that CI can prevent PD-related biofilm formation and associated pathogenic outcomes.

In this study, the CI–dextran mixture exhibited a pH of 7.4, which falls within a range unlikely to exert bactericidal effects or significantly alter bacterial physiology. Although pH is known to influence bacterial adhesion and biofilm formation, particularly for anaerobic periodontal pathogens, the measured value indicates that the inhibitory effects observed in our assays are not attributable to acidity or alkalinity of the mixture. This supports the conclusion that the reduction in biofilm formation is primarily due to the functional properties of cyclodextran, rather than pH-driven changes in the local environment.

One key observation in this study is that CI had limited direct bactericidal effects. While *P. gingivalis* exhibited a slight decrease in viability at the highest CI concentrations, *P. gulae* growth remained largely unaffected. This finding is consistent with previous studies in which CI primarily inhibited glucan synthesis rather than directly killing bacteria. The minimal cytotoxicity against the pathogens is an advantage in preventive applications, as it reduces the risk of disrupting the oral microbiome and promotes selective inhibition of pathogenic biofilm formation. Despite limited bactericidal effects, CI significantly inhibited biofilm formation by both *P. gulae* and *P. gingivalis*. Fluorescence microscopy using LIVE/DEAD staining showed thinner and less dense biofilm structures, while electron microscopy revealed disrupted biofilm architecture and reduced bacterial aggregation. Quantitative analysis confirmed a dose-dependent reduction in biofilm mass. These observations suggest that CI interferes with the initial stages of bacterial adherence and glucan-mediated extracellular matrix development, which are critical for biofilm stability and persistence.

While the biofilm quantification assay used in this study (crystal violet–based method) effectively measured total biofilm biomass, it did not provide information on biofilm architecture, such as thickness, density, or three-dimensional structure. Thus, although the results clearly demonstrated a dose-dependent inhibitory effect of CI on biofilm formation, the underlying structural alterations within the biofilm matrix remain to be elucidated. Future studies incorporating confocal laser scanning microscopy (CLSM) will be valuable to visualize the spatial organization of Porphyromonas biofilms and determine whether CI alters biofilm morphology in addition to reducing biomass. In this study, fluorescence microscopy and SEM provided qualitative confirmation of reduced biofilm formation; however, these observations were not subjected to statistical or semi-quantitative image analysis. The micrographs consistently showed decreased biofilm coverage and surface density with increasing CI concentrations, but parameters such as biofilm coverage area or mean fluorescence intensity were not quantified. Future work will include semi-quantitative image analysis using dedicated software (e.g., ImageJ or COMSTAT) to complement biomass assays and provide statistical validation of CI's effects on biofilm structure.

Biofilm formation by periodontal bacteria is largely dependent on the synthesis of extracellular polysaccharides, particularly insoluble glucans (mutans) (18). These glucans promote bacterial adhesion to tooth surfaces and provide structural integrity to biofilms, creating a protective niche that facilitates bacterial survival and virulence (19). In this study, CI treatment significantly and dose-dependently reduced insoluble glucan production in both *P. gulae* and *P. gingivalis*. This decrease was closely correlated with diminished biofilm mass, indicating that CI interferes with the biochemical processes governing glucan synthesis. Previous reports have demonstrated that cyclodextran directly inhibits glucosyltransferase (Gtf) activity in *Streptococcus mutans* by competitively binding to the enzyme's acceptor site, preventing elongation of the α-1,6-glucan chain (8, 20–22). The parallel reduction of glucan content and biofilm formation observed in the present study provides indirect mechanistic validation that CI may act through a similar pathway in Porphyromonas species. Although direct enzymatic assays were not performed here, these findings strongly support a model in which CI inhibits Gtf-mediated polymerization of glucose into insoluble glucans. By targeting this foundational step in matrix formation, CI disrupts the extracellular scaffold essential for bacterial adhesion and persistence, thereby weakening biofilm stability and pathogenic potential.

Halitosis is a clinically relevant symptom of PD, often resulting from the bacterial metabolism of sulfur-containing amino acids that generate VSCs such as H₂S and CH₃SH (10). These compounds not only contribute to malodor but also exacerbate tissue inflammation and destruction. Our results demonstrate that CI significantly reduced the production of H₂S and CH₃SH by both *P. gulae* and *P. gingivalis* in a dose-dependent manner. Importantly, CI did not directly neutralize methyl mercaptan in short-term incubation experiments, indicating that the decrease in VSCs is likely due to inhibition of bacterial metabolic activity within biofilms rather than a direct chemical deodorizing effect. By preventing biofilm maturation and glucan synthesis, CI indirectly reduces the metabolic output of pathogenic bacteria, thereby mitigating halitosis and associated tissue damage.

The inflammatory response in PD is mediated largely by host immune cells, including macrophages, which release proinflammatory cytokines such as IL-1β and IL-6 in response to bacterial components and biofilm-derived signals (23). These cytokines promote gingival inflammation, osteoclast activation, and bone resorption, contributing to disease progression (24). In this study, co-culture of macrophages with *P. gulae* or *P. gingivalis* significantly increased IL-1β and IL-6 secretion. CI treatment suppressed cytokine production in a dose-dependent manner without inducing cytotoxicity. These findings suggest that by inhibiting biofilm formation and bacterial virulence factor production, CI reduces the stimuli that trigger macrophage activation and proinflammatory signaling. Thus, CI may confer dual protective effects: directly preventing biofilm-associated bacterial activity and indirectly modulating host immune responses. The cytokine analysis in this study focused on a single 24 h stimulation period, which was chosen based on preliminary findings indicating maximal cytokine secretion within this timeframe (25, 26). While the present study suggests that CI's anti-inflammatory effects primarily arise from the suppression of bacterial biofilm and glucan formation, the possibility of direct modulation of macrophage signaling cannot be excluded. Given that certain polysaccharides and oligosaccharides are known to interact with pattern recognition receptors or influence intracellular signaling cascades, further studies examining NF-κB and MAPK pathway activation in macrophages exposed to CI alone would be valuable to delineate any host-directed immunomodulatory effects.

The findings of this study have several important implications for preventive dentistry in both humans and companion animals. In veterinary medicine, professional dental procedures such as scaling and root planing often require general anesthesia, which carries inherent risks, particularly in older or systemically compromised animals. Therefore, safe, non-invasive, daily home-care interventions that reduce biofilm formation and halitosis are highly desirable. CI, as a naturally derived, non-toxic compound with minimal bactericidal activity, is well-suited for such applications. Its ability to inhibit biofilm formation, reduce VSCs, and suppress inflammatory cytokine production highlights its potential as an adjunctive preventive agent in oral care products such as toothpaste, gels, or water additives for pets and humans. Furthermore, CI may complement or reduce the need for systemic antibiotics, which are often limited in efficacy due to biofilm-mediated resistance and can disrupt the commensal oral microbiota. By targeting the structural foundation of biofilms rather than bacterial viability, CI offers a selective approach that minimizes the risk of dysbiosis and antibiotic resistance. This approach is particularly relevant given the increasing global concern over antimicrobial resistance in both human and veterinary medicine.

The primary mechanism underlying CI's protective effects appears to involve inhibition of glucan-mediated biofilm formation. CI likely interferes with bacterial glucosyltransferases, enzymes that polymerize glucose units into insoluble glucans (20). This disruption prevents the establishment of a stable biofilm matrix, reducing bacterial adhesion, aggregation, and metabolic activity. The downstream effects of biofilm inhibition include decreased production of VSCs and reduced stimulation of host immune cells, ultimately mitigating halitosis and inflammation. It is noteworthy that CI's effects were dose-dependent, with higher concentrations producing greater inhibition of biofilm, glucan synthesis, and cytokine induction. Importantly, no cytotoxic effects were observed on macrophages, indicating a favorable safety profile for potential oral applications.

While this study provides compelling *in vitro* evidence for CI's anti-biofilm and anti-inflammatory properties, several limitations should be acknowledged. First, although the CI–dextran mixture was formulated to contain more than 13% CI, preparing a dextran-only vehicle control with matched viscosity and physical properties proved technically challenging. As a result, the precise contribution of dextran to the observed inhibition of biofilm formation could not be experimentally quantified. Instead, its potential influence was addressed through discussion rather than direct comparative data. Second, while ATP-based luminescence measurements provided an initial assessment of bacterial viability, more quantitative approaches such as CFU enumeration or MIC determination would further strengthen conclusions regarding the non-bactericidal nature of CI. Finally, the *in vitro* biofilm model used in this study does not fully replicate the complex environmental conditions of the oral cavity, and future studies incorporating multispecies biofilms or *in vivo* systems will be necessary to validate the broader applicability of CI. Despite these limitations, the findings provide important initial evidence supporting CI as a promising agent for targeted biofilm inhibition.

First, the controlled laboratory conditions do not fully replicate the complex microbial ecology of the oral cavity, and CI's effects on multispecies biofilms and commensal microorganisms remain to be determined. Second, the experiments were restricted to short-term co-culture (up to 24 h); thus, the long-term stability and efficacy of CI against mature or chronic biofilms require further investigation. Third, although murine and canine macrophage cell lines provided valuable insight into inflammatory modulation, *in vivo* studies are necessary to evaluate systemic and local immune responses within a physiological environment.

Future research should therefore include *in vivo* evaluations in dogs and humans to confirm CI's preventive effects on natural biofilm development, halitosis, and periodontal inflammation, as well as multispecies biofilm models to assess selectivity and off-target impacts. Formulation development is also needed to optimize CI delivery in oral care applications, ensuring stability, bioavailability, and user compliance. In addition, long-term safety and efficacy studies should examine repeated administration and potential impacts on the oral microbiome.

Finally, this study used CI–dextran formulations provided by WELLNEO SUGAR Co., Ltd., the industrial sponsor. Although all experiments were performed and analyzed independently, potential bias associated with material sourcing and funding should be transparently recognized. Independent synthesis, blinded analyses, and *in vivo* validation will be essential to confirm reproducibility and establish the clinical relevance of CI as a safe and effective preventive strategy for periodontal disease in both humans and companion animals.

In summary, this study demonstrates that cyclodextran effectively prevents periodontal disease-related pathogenic processes by inhibiting biofilm formation, reducing volatile sulfur compound production, and suppressing proinflammatory cytokine induction. CI exerts minimal bactericidal activity, highlighting its selective mechanism of action that targets biofilm rather than bacterial viability. These findings suggest that CI has strong potential as a preventive agent for PD in both humans and companion animals, offering a non-invasive, safe, and targeted approach to oral care. Future clinical trials are warranted to validate these effects *in vivo* and to explore its application in daily oral hygiene regimens.

## Data Availability

The datasets presented in this study can be found in online repositories. The names of the repository/repositories and accession number(s) can be found in the article/Supplementary Material.
